# Defects of the Eye and Methods of Test

**Published:** 1913-09-27

**Authors:** 


### THE SCHOOL DOCTOR.

### Defects of the Eye and Methods of Test.

These are among the most frequent that the school doctor will meet with, and a little attention paid to eyes as a specialty will be amply repaid by the increased facility in diagnosis which it brings. Ordinarily the nurse tests vision by means of a Snellen's test-card. The details of such testing need not be explained, as they are sufficiently well known. It may, however, be pointed out that the cards are often the worse for wear, dirty, and almost illegible. In that case the results are merely approximate and often misleading. A good light is sometimes difficult to obtain, especially on a murky day. By hanging the card under a gas lamp, and arranging the blackboard so as to act as a reflector, additional illumination may be secured in such cases. Sometimes it is an advantage to draw with a broad piece of coloured chalk red or blue lines underneath the letters, so as to show up the latter. The best test-cards are those with a white enamelled surface, which can be cleaned by wiping with a wet cloth; but these are comparatively expensive, and are not usually supplied.

Normal vision (6/6), as tested by the nurse, may be passed at once unless there is reason to suspect eye strain, when a more searching examination is necessary. Everything over this, however, should be tested separately by the school doctor himself. In the infant classes, owing to the fact that the children hardly know their letters, vision tests are difficult, and are not usually insisted upon. In such cases special test-cards must be used. The E type, in which the letter is represented in various attitudes—upside down, sideways, and correctly—and the child is given a letter E and told to hold it in the position of the letter pointed out to him on the card, is a useful form of test type. Modifications, in which small figures of animals, diagrams, and circles are represented on the card, are more troublesome to use, and do not seem to answer as well. The testing of the vision in children who are mentally deficient or dull is often a very laborious and troublesome process, and in certain cases it is easier and quicker to test them by retinoscopy.

When a child has been found to have his vision 9/9 or above, the question arises as to what should be done. As a general rule it may be taken that vision registered as 12/12 or higher demands treatment, and a card should be given accordingly. In the junior classes 9/12 or slighter degrees of astigmatism may be left for further examination in six months' time, as the vision considerably improves in some cases; when no improvement is registered at the second examination the child should be sent for treatment. The importance of eye strain as a defect needing treatment cannot be too fully impressed upon parents and teachers alike.

It is often a matter of considerable difficulty to get parents to see that the child needs treatment. Some parents seriously object to their children wearing spectacles; they state that they themselves suffered from "weak eyes" in their youth, and never found the defect very troublesome, and, therefore, consider that their children, too, need no treatment.

### The Parents' Point of View.

The prevalent view among parents is that a child will "grow out" of a squint. A highly interesting and instructive synopsis of popular superstitions with regard to the eyes is given by the School Medical Officer for the Acton Authority in his last report, in which this superstition is commented upon. When it is considered that a squinting eye invariably becomes the weaker eye, and may ultimately become quite blind, the importance of insisting upon treatment is obvious. Most education authorities now have made admirable arrangements for supplying glasses and for securing treatment for cases of defective vision. Many, too, have on their medical staff a special ophthalmologist, to whom certain cases that present difficulty may be referred for a second opinion. In these circumstances there is little excuse for neglecting a child with imperfect vision. Spectacles are procurable for from 2s. 9d. to 3s. 6d.; in special cases, where special lenses are ordered, this sum may have to be considerably augmented. Such cases should be specially recommended to the Care Committees if the parents cannot afford to buy the glasses. The school doctor should carefully test the child's vision when the glasses have been procured. Sometimes parents go to the nearest optician, and (although opticians as a rule do their work very well) the results are not always satisfactory. Similarly, unsatisfactory prescriptions are sometimes given by hospitals or private practitioners, and in every case, therefore, the glasses should be tested and approved or rejected as the case may be. A rejection means a considerable amount of trouble to all concerned, and no one who knows anything at all about school medical inspection will assert that school doctors are prone to reject glasses as unsuitable, since such rejection gives them an added amount of work and trouble.

The tendency, indeed, is all the other way—to accept treatment given by a hospital or practitioner as thoroughly adequate, and to pass the glasses merely because the child has had a mydriatic and has been seen at an eye department of some hospital. When the parent is told that the glasses are unsuitable, much tact is generally demanded to induce him or her to have a new pair made or a new examination of the child's eyes. Such should be insisted upon, however, in every case where the school doctor finds that the glasses are unsuitable, either as regards the lenses or as regards the frame-work. When the frame is bad the child soon discards the glasses, because he "sees better without them," or "they hurt." Constant supervision is demanded in such cases unless a little preliminary care is taken to see that the glasses are really suitable.

Some schools now have examination rooms—and in course of time probably every school will have its specially arranged room for medical inspection—with blinds that can be pulled down so as to darken the room. Where such exist, laryngoscopic and ophthalmoscopic examinations may be indulged in when the inspector finds time for such work.

Diseases of the eyelids and of the conjunctiva are much more common. Of these the commonest is blepharitis, which is largely a " dirt disease," and in school children is readily curable by home treatment steadily and methodically applied. A particularly bad form is seen in verminous children, who, as a preliminary to medical treatment, should be sent to attend a cleansing station a few times. Purulent conjunctivitis, or blight, as its various forms are commonly called, is easily diagnosable, and should be promptly dealt with. Children suffering from it should be at once excluded, and should not be allowed to return to class until the condition has been cured. Children who attend " picture shows " sometimes show symptoms of eye strain in a marked degree, and examination will generally prove that they suffer from slight degrees of astigmatism or myopia, necessitating the wearing of glasses.

